# Micro-Doppler Signal Time-Frequency Algorithm Based on STFRFT

**DOI:** 10.3390/s16101559

**Published:** 2016-09-24

**Authors:** Cunsuo Pang, Yan Han, Huiling Hou, Shengheng Liu, Nan Zhang

**Affiliations:** 1 National Key Laboratory for Electronic Measurement Technology, North University of China, Taiyuan 030051, China; hanyan@nuc.edu.cn (Y.H.); hou_huiling@126.com (H.H.); 2 School of Information and Electronics, Beijing Institute of Technology, Beijing 10081, China; henry@bit.edu.cn; 3 The Second System Design Department of Second Research Academy of CASIC, Beijing 100854, China; matt@bit.edu.cn

**Keywords:** Micro Doppler frequency, short-time fractional order Fourier transformation, multi-order matching, order selection

## Abstract

This paper proposes a time-frequency algorithm based on short-time fractional order Fourier transformation (STFRFT) for identification of a complicated movement targets. This algorithm, consisting of a STFRFT order-changing and quick selection method, is effective in reducing the computation load. A multi-order STFRFT time-frequency algorithm is also developed that makes use of the time-frequency feature of each micro-Doppler component signal. This algorithm improves the estimation accuracy of time-frequency curve fitting through multi-order matching. Finally, experiment data were used to demonstrate STFRFT’s performance in micro-Doppler time-frequency analysis. The results validated the higher estimate accuracy of the proposed algorithm. It may be applied to an LFM (Linear frequency modulated) pulse radar, SAR (Synthetic aperture radar), or ISAR (Inverse synthetic aperture radar), for improving the probability of target recognition.

## 1. Introduction

For a complicated movement target with a translational movement, the target itself or the structure it carries may also have rotation, precession, nutation, or other micro movement features. These features could be used to help determine the “identity” of the target. The feature of micro-Doppler was firstly found in a coherent laser radar system. With a wavelength ranging from 1/100,000 to 1/10,000 of conventional radar’s wavelength, laser radar provides higher phase/frequency sensitivity, which is beneficial to the measurement and analysis of fine movement features of the targets [1,2,3,4,5,6,7,8,9]. In 2006, Chen et al. introduced micro-Doppler into conventional radar and pointed out that Doppler signals of the target echoes are mostly multi-component time-varying signals. In order to obtain micro movement features of the target, the most straightforward approach is to use the time spectrum analysis [1,2]. Time-frequency algorithms currently used by researchers mainly include linear time-frequency technique and second order Cohen’s class time-frequency technique. Linear time-frequency distribution has no cross terms but its time-frequency resolution is low. By contrast, second-order Cohen class time-frequency distribution offers a higher time-frequency resolution but its downside is the cross terms being tainted by multi-component signals [4,10,11,12,13,14,15,16,17,18].

As a general form of Fourier transformation, FRFT (fractional order Fourier transformation) has attracted extensive attention in the signal processing community in recent years thanks to its good aggregation when dealing with linear frequency modulation (LFM) signals, a property critical for dealing with non-stationary time-varying signals [19,20,21,22,23,24,25]. More importantly, FRFT—as a means of one-dimensional linear transformation—has no cross-term influence when dealing with multi-component LFM signals. However, when dealing with Doppler time-varying signals, FRFT as a global transformation technique cannot unveil the time-varying features of the target signal. Ref. [26,27,28,29,30] investigates the performance of STFRFT (short-time fractional order Fourier transformation) in resolving time-frequency signals by applying the principle of STFT (short-time Fourier transformation) to time-frequency analysis [16,31,32,33,34]. Ref. [27] uses STFRFT for separation of micro-Doppler features of the target signals from the clutter signals of a sea clutter background. These studies demonstrate the efficiency of STFRFT in micro Doppler signal analysis. However, the research work is not sufficient on improving the time-frequency resolution performance when dealing with multi-component signals, as well as on improving the speed of the algorithm.

This paper studies the STFRFT algorithm from both theoretical and application aspects. First, the STFRFT-based time-frequency analysis method is reviewed, from which a theoretical time-frequency resolution and a quick calculation method are proposed. Then, the analysis method of multi-order STFRFT is developed based on multi-component micro Doppler signal. Finally, experiment data are used to demonstrate the high time-frequency resolution performance of STFRFT when dealing with multi-component micro Doppler signal.

## 2. STFRFT-Based Time-Frequency Analysis Technique

### 2.1. Basic Principle of STFRFT

As with STFT, STFRFT is also a kind of windowed transformation; alternatively, it may be interpreted as an expansion of a signal on the basis of time-fractional order domain frequency location function. For a given signal, the *p*-order short-time fractional Fourier transformation is [26]:

(1) $$STFRFT_{p}(t,u)=\int_{-\infty }^{+\infty }s(\tau )\omega (\tau -t)K_{p}(\tau ,u)d\tau ,\;p=\frac{2}{\pi }\alpha$$

where, $K_{p}\left(u,t\right)$ is the kernel function.

Different window functions have different time-frequency aggregation; Ref. [26] points out that Gauss window has a high aggregation in time-frequency domain and its window function is:

(2) $$\omega (t)=A\exp(-c\frac{t^{2}}{2})$$

where, $A=\frac{1}{\sqrt{2\pi }\delta_{t}}$ is magnitude, $c=1/\delta_{t}^{2}$, $\delta_{t}$ represents 3 dB width of Gauss signal in time domain, i.e., time domain resolution.

**Figure 1:**
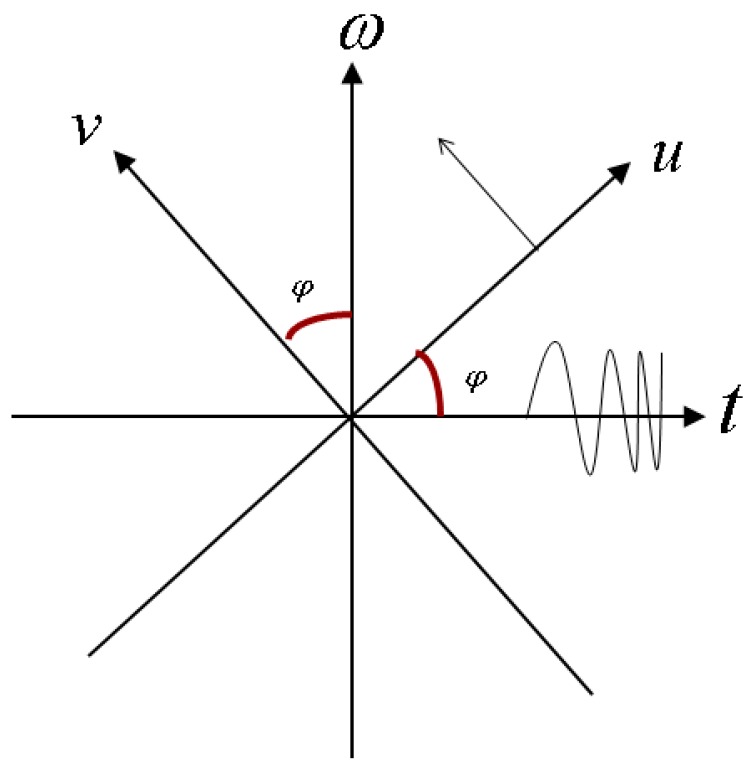
Relations between FRFT and FT time-frequency in rotational planes.

STFRFT firstly divides the target signal into a series of time intervals and then performs FRFT upon each interval of signal. Signal processing using FRFT is equivalent to rotating the time-frequency axis of Fourier transformation (FT), i.e., the signal is observed in a new coordinate system, as shown in Figure 1.

According to the time-frequency transformation relation between FRFT and FT (Figure 1), it is possible to obtain the following relation between FRFT frequency information and FT time-frequency information:

(3) $$\delta_{u}^{2}=\delta_{t}^{2}\cos^{2}\varphi +\delta_{\omega }^{2}\sin^{2}\varphi$$

where, $\delta_{t}$ represents the time resolution of the signal in FT domain, and frequency domain resolution is $\delta_{w}=1/\delta_{t}$.

For the purpose of digital processing, let $\delta_{t}$ have *m* discrete sampling points in time domain, δ_ω_ have N/2πm discrete sampling points in frequency domain. Substituting them into Equation (3) gets the following discrete form:

(4) $$\delta_{u}^{2}=\frac{N^{2}}{4\pi^{2}\delta_{t}^{2}}\sin^{2}\varphi +\delta_{t}^{2}\cos^{2}\varphi$$

where, the total number of sampling points *N* depends on Gauss window length.

Equation (4) gives the time-frequency relation in STFRFT domain for any signal. Additionally, for LFM signals and when rotation angle φ matches the frequency modulation rate of the detected signal, the time-frequency resolution becomes the largest and then Equation (4) is simplified into:

(5) $$\delta_{u}^{2}=\frac{N^{2}}{4\pi^{2}\delta_{t}^{2}}$$

### 2.2. Quick Order Selection for STFRFT Domain Transformation

STFRFT’s computation load is much greater than that of STFT, mainly due to optimum order selection for fractional order domain. For this reason, it hardly meets the requirement for practical application. A technique is proposed in this section to process a short time and actual signals with continuity in both time and space. Specifically, a preliminary order prediction is made using Lagrange’s interpolation polynomial, and fine searching is performed on the basis of the prediction. This technique improves computation efficiency.

#### 2.2.1. Order Selection

If short time signals centered on $t_{0},t_{1},\cdots t_{L}$ have an order of $p(t_{0}),p(t_{1}),\cdots p(t_{L})$ respectively when the maximum peak occurs in the FRFT domain, and if p(t) in a short interval can be expressed in an (*n* + 1) order polynomial, the Lagrange polynomial of p(t) in any small neighborhood $t_{0}\in \left(t_{1},t_{2}\right)$ may be written as:

(6) $$p(t)\approx \sum_{k=0}^{L}p(t_{k})\ell_{k}(t)$$

In Equation (6), each basis function $\ell_{k}(t)$ can be written as:

(7) $$\ell_{k}\left(t\right)==\prod_{j=0,j\neq k}^{L}\frac{x-x_{j}}{x_{k}-x_{j}}$$

Substituting Equation (7) into Equation (6) and after discretization, we get:

(8) $$p\left(t_{i}\right)\approx \sum_{k=0}^{L}p(t_{k})\prod_{j=0,j\neq k}^{L}\frac{t_{i}-t_{j}}{t_{k}-t_{j}}$$

where, $t_{i}$ is the *i*th discrete time interval of the signal.

Generally, *L* in Equation (8) takes a value of 2 or 3, where, the *L* is valuated with consideration given to the trade-off between the computation load and the effectiveness of the algorithm. With the proposed technique, the signals are initially divided into several time intervals for window processing, and then the windowed signals are processed by FRFT. As FRFT is most effective in analyzing linear frequency modulation (LFM) signals (i.e., secondary phase signals), it is desirable to have windowed signals with a shorter time duration such that the secondary phase relation is best satisfied. It is also assumed that adjacent signals are continuously derivable as a function of time. Therefore, the optimum order *p* of the signals in FRFT domain does not vary much from one signal to an adjacent one. Also, a larger *L* involves greater computation load and so, for the benefit of overall efficiency, *L* is typically set equal to 2 or 3.

When *L* = 2, Equation (8) can be reduced to:

(9) $$p\left(t_{i}\right)\approx p(t_{0})\ell_{0}(t)+p(t_{1})\ell_{1}(t)+p(t_{2})\ell_{2}(t)$$

where, $\ell_{0}(t)=\frac{\left(t_{i}-t_{1})(t_{i}-t_{2})(t_{i}-t_{3}\right)}{\left(t_{0}-t_{1})(t_{0}-t_{2})(t_{0}-t_{3}\right)}$; it is similar for $\ell_{1}(t)$ and $\ell_{2}(t)$.

For practical digital processing, Equation (9) firstly estimates the value of order. Searching is thus restricted within a small range, hence enhancing the computation efficiency of STFRFT algorithm.

#### 2.2.2. Analysis of Frequency Resolution Error

When the transformation order matches the LFM signal frequency modulation rate, resolution of FRFT frequency reaches the maximum. However, for an LFM signal with an unknown frequency modulation rate, there certainly exists an error in order matching because of difference in searching step interval Δp. The following paragraphs analyze the influence of Δp on FRFT domain frequency resolution.

Let $p_{0}$ be the transformation order for signal matching, $p_{0}^{\prime }$ be the actual transformation order, and $p_{0}-p_{0}^{\prime }$ be the transformation order error. From Equations (4) and (5), the frequency resolution error $\sigma_{\Delta p}$ caused by Δp is:

(10) $$\begin{array}{ll} \sigma_{\Delta p} & =\sigma_{p_{0}}-\sigma_{p_{0}^{\prime }}\\ & =\{\begin{array}{ll} 0 & \Delta p=0\\ \frac{N-\sqrt{N^{2}\sin^{2}(\frac{p_{0}^{\prime }}{2}\pi )+4\pi^{2}\sigma_{t}^{4}\cos^{2}(\frac{p_{0}^{\prime }}{2}\pi )}}{2\pi \delta_{t}} & \Delta p\in (0,1] \end{array} \end{array}$$

**Figure 2:**
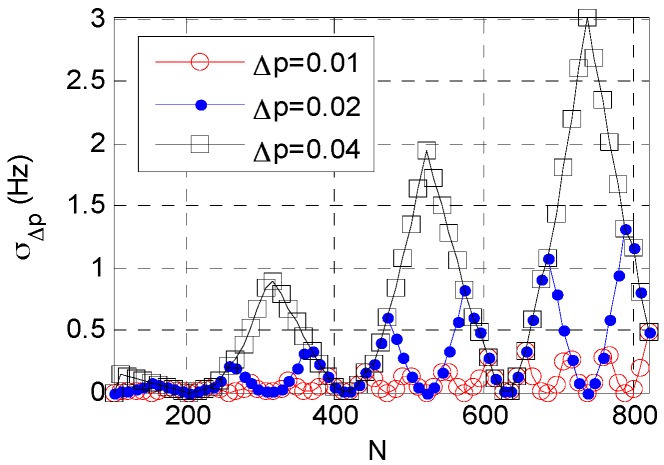
Relation between $\sigma_{\Delta p}$ and N.

**Figure 3:**
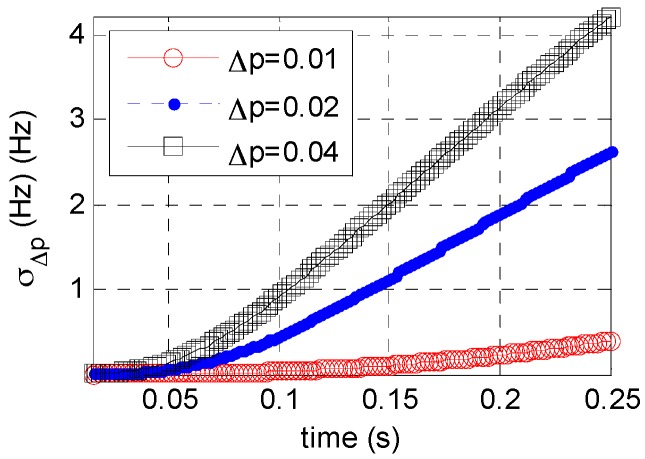
Relation between $\sigma_{\Delta p}$ and $\sigma_{t}$.

Equation (10) suggests that error $\sigma_{\Delta p}$ is dependent on $\sigma_{t}$, N, and Δp. Figure 2 and Figure 3 show the relation between $\sigma_{\Delta p}$ and N or $\sigma_{t}$.

Figure 2 shows that at a constant Δp, $\sigma_{\Delta p}$ varies periodically with an increasing N, but generally a larger N corresponds to a larger $\sigma_{\Delta p}$. Figure 3 shows that: at a constant Δp, $\sigma_{\Delta p}$ increases with a decrease in time resolution. For a given accumulated time, a larger Δp leads to a larger $\sigma_{\Delta p}$. This finding has practical implications, because when accumulated time increases or time resolution decreases the searching step interval of order should be shortened; otherwise, the searching step interval should be increased. This adaptive adjustment is critical for order step searching and helps reduce the computation load.

### 2.3. STFRFT’s Time-Frequency Analysis Capability of Time-Varying Signal

#### 2.3.1. One-Component Signal Analysis

If noise and clutter influence are neglected, the polynomial phase signal model becomes:

(11) $$s(t)=rect(\frac{t}{T})\cdot \exp j2\pi [f_{0}t+\varphi_{e}(t)]$$

**Figure 4:**
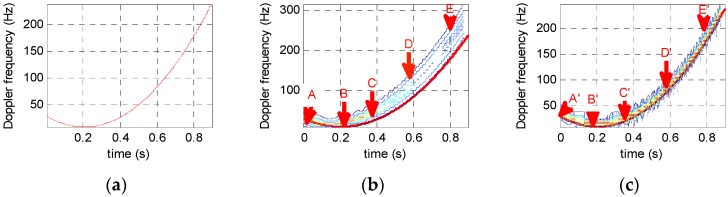
Comparison of time-frequency analysis for different algorithms. (**a**) Theoretical time-frequency relation results; (**b**) STFT results; (**c**) STFRFT results.

Figure 4a shows the theoretical time-frequency spectrum when T=1 s, $f_{s}=1024\mathrm{Hz}$, $f_{0}=10\mathrm{Hz}$, and polynomial phase signal $\varphi_{e}\left(t\right)=160t^{3}-100t^{2}+20t$. Figure 4b,c show the time-frequency analysis results of STFT and STFRFT respectively. For this experiment, the Gauss window function width has 128 sampling points and the order searching interval is 0.01.

When variation in signal frequency is small, STFT and STFRFT have similar frequency resolution, as indicated by points A and B in Figure 4b,c, respectively. However, with the increase in frequency variation, the resolution ratio for these two techniques becomes larger within the same time window width.

Furthermore, to better compare the time-frequency resolving results shown in Figure 4 produced by different techniques, the time-frequency resolutions of the five points (A, B, C, D and E) on the time-frequency curves in Figure 4b,c, as generated with a same window width, are compared in Table 1.

It is evident from Table 1 that when the signal frequency variation is small, STFT and STFRFT have similar frequency resolution, as indicated by points A and B in the figure. However, with an increasing frequency variation and within the same time window width, the resolution ratio for the two techniques becomes larger, reaching 2 at point E. This ratio tends to grow as frequency variation increases.

#### 2.3.2. Computation Load Analysis

Using as an example the polynomial phase signal $\varphi_{e}\left(t\right)=160t^{3}-100t^{2}+20t$ in Equation (11), Table 2 compares Equation (11) technique with Ref. [26] (i.e., exhaustive search technique) for their order searching computation load in signal detection. Equation (11) technique uses a Gauss window width of 128, a time domain resolution of 20, and a search step of 0.01. The prediction order *L* is 2 and 3 respectively, and the SNR (signal-to-noise ratio) is −4 dB, −6 dB, −8 dB respectively.

It is clear from Table 2 that Technique 1 takes the longest searching time and Technique 2 or 3 takes far less searching times, reducing by 90%. However, it is found that the estimate error of Equation (9) increases with a dropping SNR, so a higher computation load is required. Therefore, in practical application it is necessary to consider SNR influence on computation efficiency.

#### 2.3.3. Analysis of a Sinusoidal Signal

**Figure 5:**
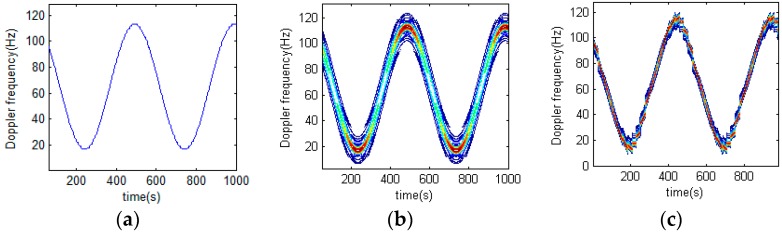
Analysis of sinusoidal time-frequency signal using different techniques. (**a**) Original signal; (**b**) Result of STFT processing; (**c**) Result of STFRFT processing.

Section 2.3.1 analyzes a polynomial phase signal by use of STFRFT. Here is a description of the performance of STFRFT in processing sinusoidal signals. Figure 5a presents the time-frequency relation of a certain signal, whose form is close to that of a sinusoidal signal. Figure 5b,c shows the result of STFT processing and STFRFT processing respectively. For an otherwise identical parameter setting, STFT produces the best time-frequency result when the window length is 32, and STFRFT produces the best time-frequency result when the window length is 64, because for STFT the signal in the window is approximately sinusoidal while for STFRFT the signal in the window is approximately LFM, which consequently has a longer window. Additionally, it is obvious from Figure 5b,c that STFRFT fares better in terms of time-frequency resolution than STFT.

#### 2.3.4. Multi-Component Signal Analysis

**Figure 6:**
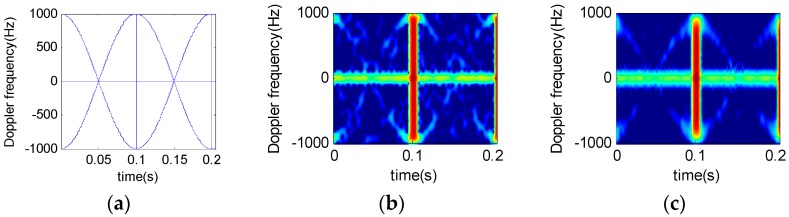
Micro-doppler time-frequency spectrum for two-blade helicopter. (**a**) Theoretical time-frequency relation results; (**b**) STFT results; (**c**) STFRFT results.

**Figure 7:**
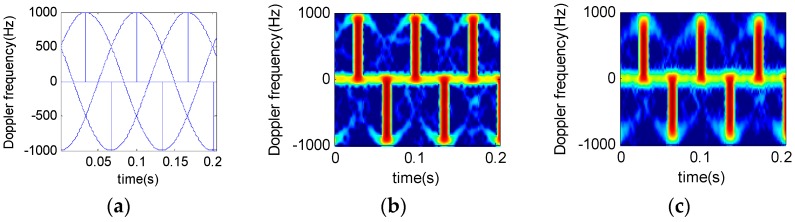
Micro-doppler time-frequency spectrum for three-blade helicopter. (**a**) Theoretical time-frequency relation results; (**b**) STFT results; (**c**) STFRFT results.

Suppose that a radar system works at S-band and the wavelength is 0.1 m, the pulse interval is 200 ms, the accumulated pulse number is 2048, the helicopter has two blades, at a rotation rate of 5 Hz/s. The distance between the blade root and the rotor wing center is 3 m. Figure 6 and Figure 7 show the time-frequency results of analysis on different rotor helicopter echoes. The short-time window length is 64 and the window function is a Gauss function.

Figure 6 and Figure 7 suggest that, under a given condition, the STFRFT time-frequency spectrum has the least clutter interference, thus offering better detection performance than the STFT technique. 

By comparing Figure 6c and Figure 7c, we find that the time-frequency resolution of STFRFT depends on the number of blades. It follows that a higher number of blades require higher resolution in time-frequency analysis algorithm of the echo signal.

## 3. Multi-Order STFRFT Time-Frequency Analysis Technique

It is known that micro-Doppler signals are composed of multiple components, which have distinct time-frequency features. Therefore, it is impractical to perform matching processing by a particular order of fractional order Fourier transformation. In consideration of this, multi-order short-time fractional Fourier transformation is proposed for signal time-frequency analysis. This technique is represented by the following expression:

(12) $$\begin{array}{ll} S(t_{p},f_{p}) & =STFRFT_{s,p}(t,u)\\ & =\int_{-\infty }^{+\infty }s(\tau )w(\tau -t)K_{p}(\tau ,u)d\tau ,\;p=0,\;\Delta p,\ldots Q\Delta p \end{array}$$

where, *Q* is the total number of orders, Δp the interval of fractional order.

After processing constant false alarm by Equation (12), we get:

(13) $$\left|S(t_{p},f_{p})\right|=\begin{array}{cc} \left|S(t_{p},f_{p})\right|, & \frac{\left|S(t_{p},f_{p})\right|}{\delta }\geq th\\ 0, & \frac{\left|S(t_{p},f_{p})\right|}{\delta }<th \end{array}$$

where, δ is the noise variance, $th=\sqrt{2\ln(1/P_{fa})}$ the detection threshold, and $P_{fa}$ the CFAR.

Different time-frequency spectra obtained from Equation (13) are processed using data fusion technique, and the following expression is derived:

(14) $$S(t,f)=\sum_{q=1}^{Q+1}\left|S(t_{p},f_{p})\right|,\;p=0,\;\Delta p,\cdots Q\Delta p$$

where, $S(t_{p},f_{p})$ represents the distribution of micro-Doppler signal time-frequency corresponding to different orders, S(t,f) is the distribution of micro-Doppler signal time-frequency after data fusion.

In practical application and if s(t) SNR is high, Equation (14) may be reduced to:

(15) $$S(t,f)=\sum_{q=1}^{Q+1}|STFRFT_{s,p}\left(t,u\right)|,\;p=0,\;\Delta p,...Q\Delta p$$

**Figure 8:**
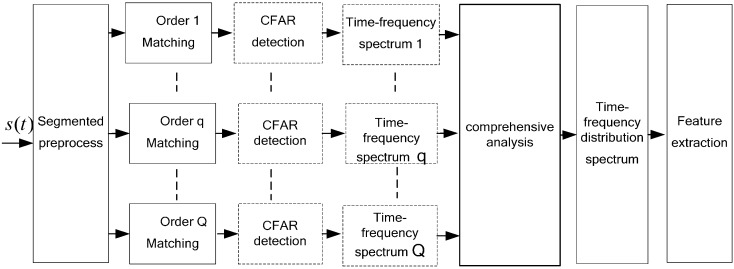
Multi-order stfrft-based micro-doppler signal time-frequency analysis diagram.

The above time-frequency analysis process, as defined in Equations (14)–(17), may be described by the processing procedure shown in Figure 8.

The comprehensive analysis of data given in Figure 8 may be processed by direct accumulation of multiple sub-time-frequency spectra. For sub-time-frequency spectra obtained from a strong clutter background, image processing techniques—such as smoothing, filtering, expansion, and etching—may be used for preprocessing before superposition processing.

## 4. Experiment Analysis

### 4.1. Actual Signals from a Rocket Projectile Target

The major parameters of the radar system used in the experiment are given in Table 3. This section focuses on analyzing the frequency spectrum variation features of the target echo signals throughout the entire flight stage.

**Figure 9:**
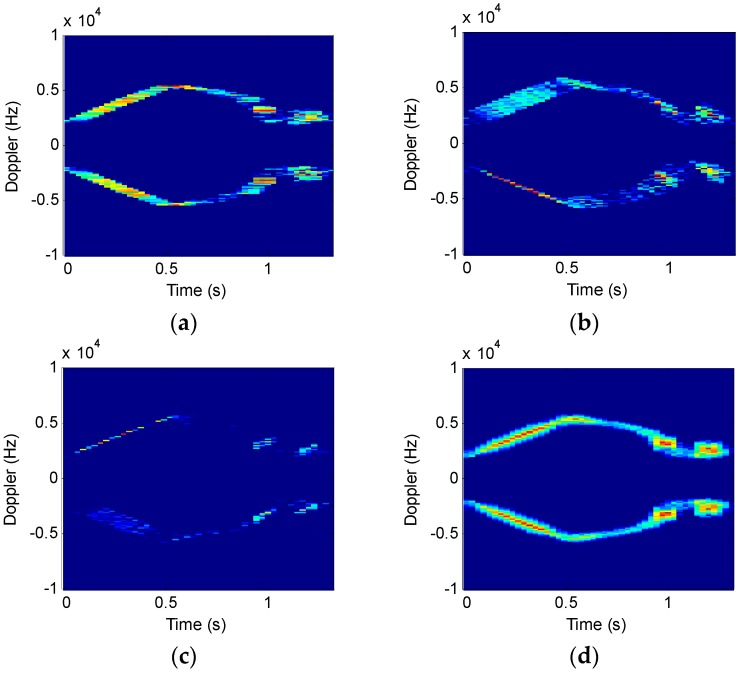
Comparison of STFT and STFRFT analysis results. (**a**) STFT results; (**b**) STFRFT (*p* = 0.93) results; (**c**) STFRFT (*p* = 1.05) results; (**d**) STFRFT results after comprehensive processing.

Figure 9 shows the analysis results of three time-frequency algorithms under different SNR backgrounds. A Gauss window is used as the window function. The window length is 4096 points and the order interval is 0.01.

As shown in Figure 9b,c, STFRFT can obtain part signal of high time-frequency resolution when the order is 0.93 and 1.05. After comprehensive processing of all spectra, STFRFT (Figure 9d) is found to have a better image than STFT (Figure 9a). 

**Figure 10:**
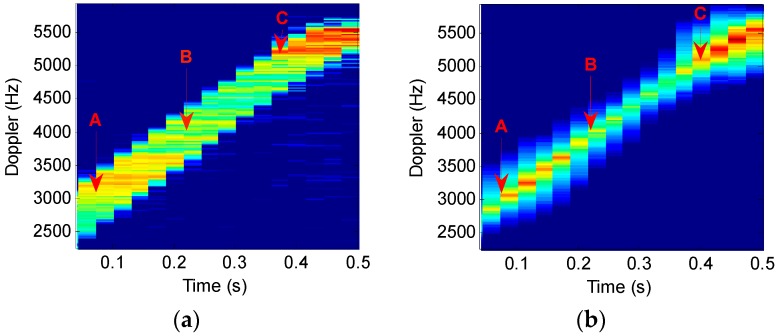
Comparisons of STFT and STFRFT local time-frequency analysis results. (**a**) STFT; (**b**) STFRFT.

To underscore the meaningful improvement in time-frequency resolution performance, Figure 10 compares, in an enlarged view, the portions of the STFT and STFRFT time-frequency images in Figure 9a,d. Points A, B, and C in Figure 10a,b are generated in the same time interval; the three points in Figure 10a occupy 158, 189, and 235 frequency units respectively, while the three points in Figure 10b occupy 124, 151, and 191 frequency units respectively. Drawing on [13], we performed, by use of three-point method, Doppler frequency estimation of points A (0.1145, 3040), B (0.2577, 4468), C (0.4009, 4936) in Figure 10a and points A (0.1145, 3028), B (0.2577, 4287), C (0.4009, 4926) in Figure 10b, with the results being 0.52 Hz and 0.43 Hz respectively. Table 4 compares the time-frequency resolutions achieved by STFT and STFRFT.

Table 4 suggests that STFRFT improves on STFT by about 25% on average in time-frequency resolution, good for improving time-frequency separation of multi-component signals and parameter estimation accuracy of Doppler frequency.

Relative to STFT, the proposed STFRFT is more effective in increasing the SNR of targets because for STFRFT the window contains signal of LFM form and for STFT sinusoidal form and, with the parameters being the same for both techniques, LFM signal is longer than a sinusoidal one, thus the energy is greater after accumulation, so is the SNR.

**Figure 11:**
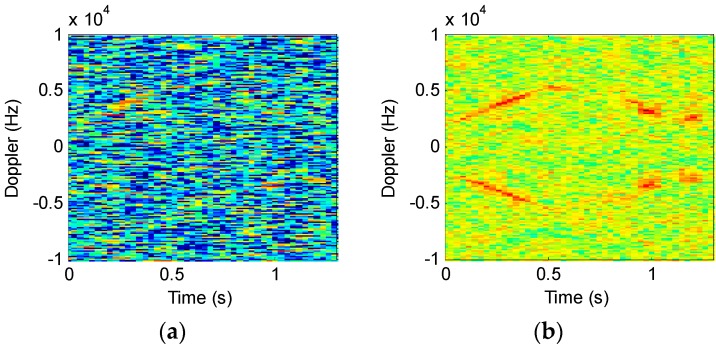
Comparison of the results after the introduction of noise. (**a**) STFT; (**b**) STFRFT.

To demonstrate the effectiveness, we introduced −5 dB Gaussian noise while analyzing the original signal shown in Figure 9; Figure 11 compares the results of the two techniques, where STFT uses a window length of 2048 and STFRFT a length of 4096. It can be seen from Figure 11 that—with a lower SNR—the detection effectiveness of STFT is degraded remarkably, as is evident in Figure 11a, where the signal of the target is hardly discernible, whereas in the case of STFRFT the signal of the target remains discernible.

### 4.2. Signals from a Real Model Helicopter Target

In this experiment, a P-band radar system was used to acquire data on a model rotor helicopter for micro movement feature analysis. The model helicopter is Align 750e; its length, height, and width are 1343, 424 and 210 mm respectively. The main rotor is 700 mm long and the main rotor diameter is 1582 mm. The tail rotor wing is 281 mm in diameter and rotates at 26–42 r/s. The propeller is of co-axial single-blade type (two blades). Table 5 lists the main parameters of the acquisition system used in this experiment.

**Figure 12:**
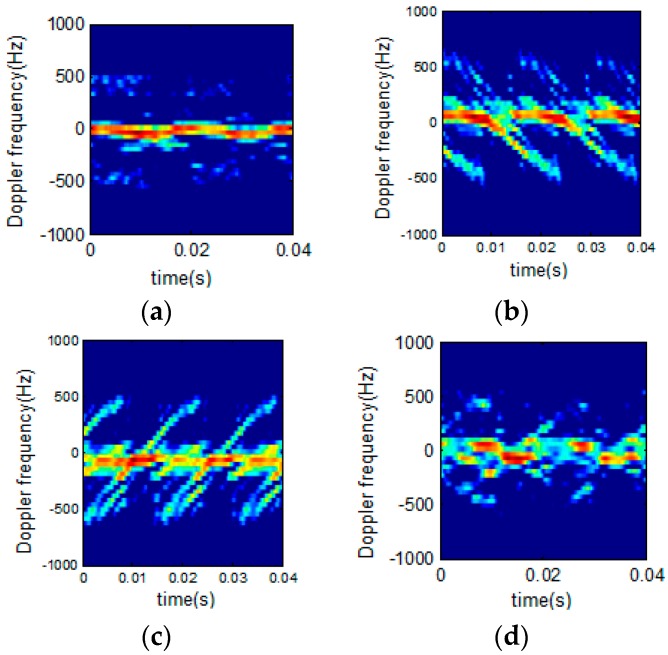
Time-frequency analysis of helicopter echo signals. (**a**) STFT results; (**b**) STFRFT (*p* = 0.9) results; (**c**) STFRFT (*p* = 1.1) results; (**d**) STFRFT results after comprehensive processing.

**Figure 13:**
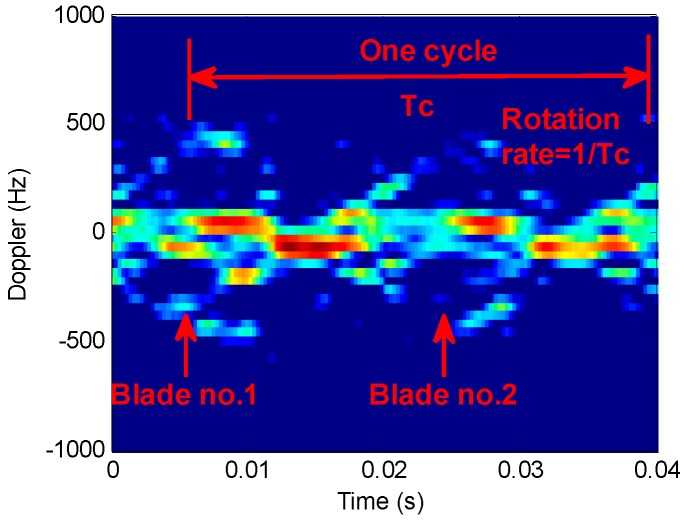
Diagram of propeller parameters.

Figure 12 shows time-frequency analysis of *r* echo signals from a two-blade rotor helicopter. The window length is 64 and the order interval is 0.1. Figure 12a indicates that STFT algorithm works well only in the bottom and the top signal time-frequency analysis. Figure 12b,c show the results when the order is not consistent. STFRFT performs better than STFT in both local signal detection and comprehensive frequency analysis. Finally, the rotation frequency of the propeller blade in Figure 12d is 35 revolutions per second, which agrees with the theoretical value of the helicopter model. Please refer to the markings of the time-frequency curve parameters in Figure 13 for the computation of the rotation rate of the propeller, where, Tc represents a rotational period of the propeller, the rotation frequency being the reciprocal of the period, i.e., 1/Tc.

### 4.3. Signals from the Bird Target

**Figure 14:**
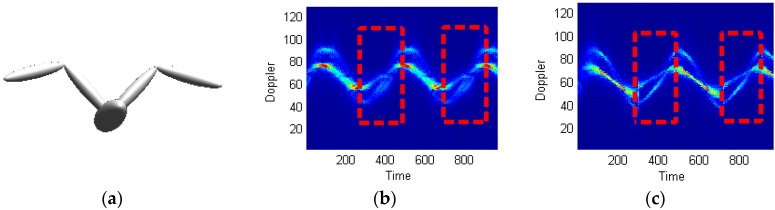
Micro Doppler time-frequency analysis of flapping wings. (**a**) Bird target model; (**b**) STFT; (**c**) STFRFT.

Figure 14 shows the bird target model in [35], the micro Doppler echo signal of which is analyzed using the proposed technique.

**Figure 15:**
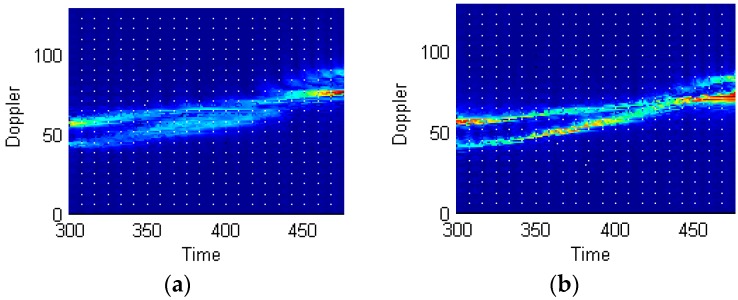
Enlarged views of portions of Figure 14. (**a**) STFT; (**b**) STFRFT.

Supposing the radar wave length is 0.03 m, the time of accumulation is 10 s, the number of accumulated impulses is 8192, the bird wing length is 1 m (both the upper and lower arms being 0.5 m long), and the flapping frequency is 2 Hz. Figure 14 shows the processing results using STFT and STFRFT and, for better revealing their differences, Figure 15 provides enlarged views of the red box areas in Figure 14. It is apparent that STFRFT performs appreciably better than STFT in time-frequency focusing.

### 4.4. Actual Fan Target Signals

To further verify micro-Doppler features of propeller type targets, fan blades were used to simulate a helicopter propeller in an experiment. 

#### 4.4.1. Dual-Blade Fan

**Figure 16:**
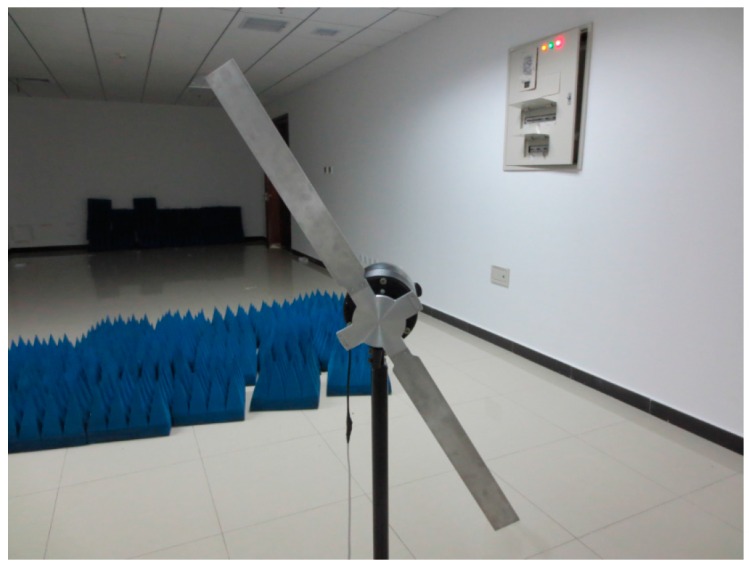
A dual-blade fan.

Figure 16 shows a self-manufactured dual-blade fan, whose blades are 30 cm long and 3 cm wide. The fan motor may produce a rotation speed up to 15 r/s. The parameters of the radar system are given in Table 6. The blue sponges in Figure 16 are absorption materials.

**Figure 17:**
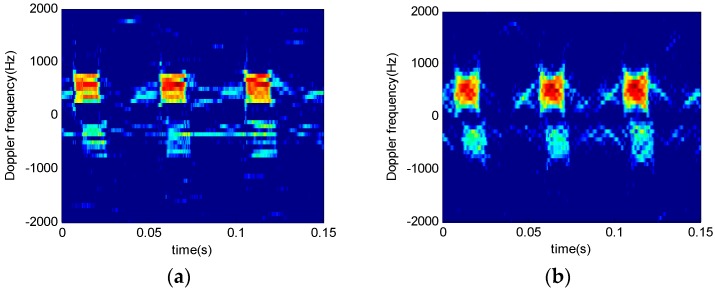
Comparative analysis of two techniques. (**a**) STFT; (**b**) STFRFT.

Figure 17a,b show the processing result using SFFT and STFRFT respectively, STFT having a window length of 16 and STFRFT a window length of 32, with the rest of the parameters being the same for both techniques. It is obvious from Figure 17a,b that STFRFT fares better in resolving time-frequency than STFT, as it reveals more time-frequency component signals.

#### 4.4.2. Three-Blade Fan

**Figure 18:**
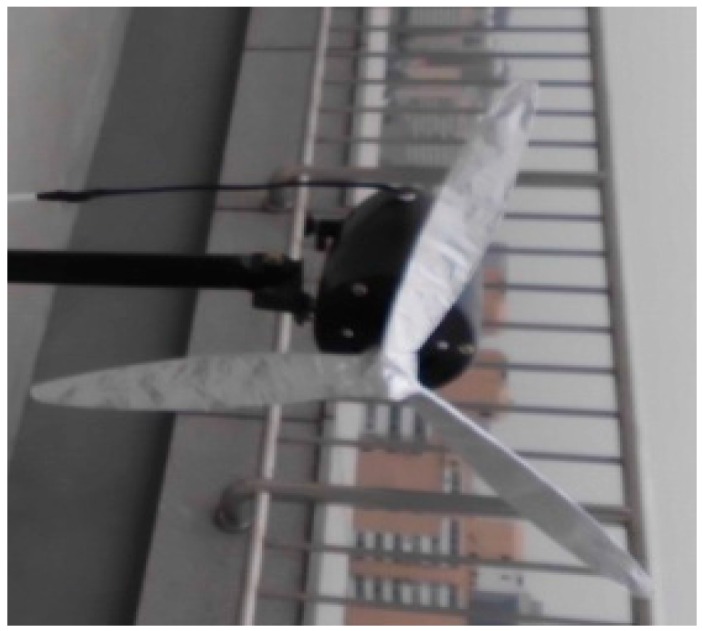
Three-blade fan.

**Figure 19:**
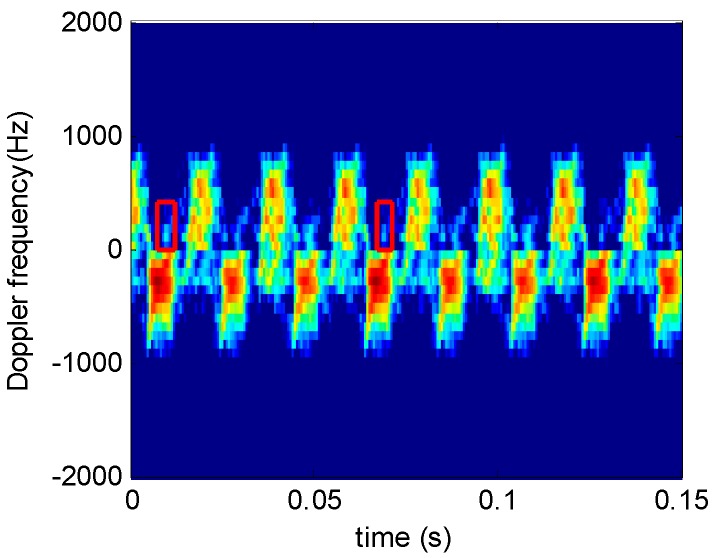
STFRFT time-frequency analysis.

The experiment fan is shown in Figure 18, and the system parameters are given in Table 6. The fan shown in Figure 18 is an industrial ox horn fan (rotation speed: 18–23 r/s); Figure 19 shows the STFRFT time-frequency analysis result of this fan, the window length being 256.

Unlike Figure 12d and Figure 17b, the increased number of blades makes it impossible to determine clearly the number of time-frequency curves from Figure 19. Alternately, a blade (shown in red boxes) on Figure 19 is found to have time-frequency features not identical to other blades. This is because the target attitude has some influence on the blades, which are located differently in space. Consequently, the micro-Doppler signals generated by different blades may have slight differences. Thus, we can use the same blade recurring cycle to estimate blade number.

The period of the time-frequency spectrum on Figure 19 (in red) cannot be extracted directly. Hence, technique of image registration is used to extract the spectrum of this period. Image registration detects and selects images with identical scenes by selecting a proper subtemplate and moving it over the entire image. When the subtemplate coincides with a portion of the entire image, the correlation coefficient reaches maximum and the period of the same image could be extracted. The correlation coefficient is:

(16) $$\begin{array}{ll} \rho & =\frac{E(S_{1}S_{2})}{\sqrt{D(S_{1})D(S_{2})}}\\ & =\frac{\frac{1}{MN}\sum_{m=1}^{M}\sum_{n=1}^{N}s_{1}(m,n)s_{2}(m,n)}{\sqrt{[\frac{1}{MN}\sum_{m=1}^{M}\sum_{n=1}^{N}s_{1}^{2}(m,n)][\frac{1}{MN}\sum_{m=1}^{M}\sum_{n=1}^{N}s_{2}^{2}(m,n)]}}\\ & =\frac{\sum_{m=1}^{M}\sum_{n=1}^{N}s_{1}(m,n)s_{2}(m,n)}{\sqrt{[\sum_{m=1}^{M}\sum_{n=1}^{N}s_{1}^{2}(m,n)][\sum_{m=1}^{M}\sum_{n=1}^{N}s_{2}^{2}(m,n)]}} \end{array}$$

where ρ is the correlation coefficient, $S_{1}$ the template image, and $S_{2}$ the entire image, and (M,N) the template image size.

**Figure 20:**
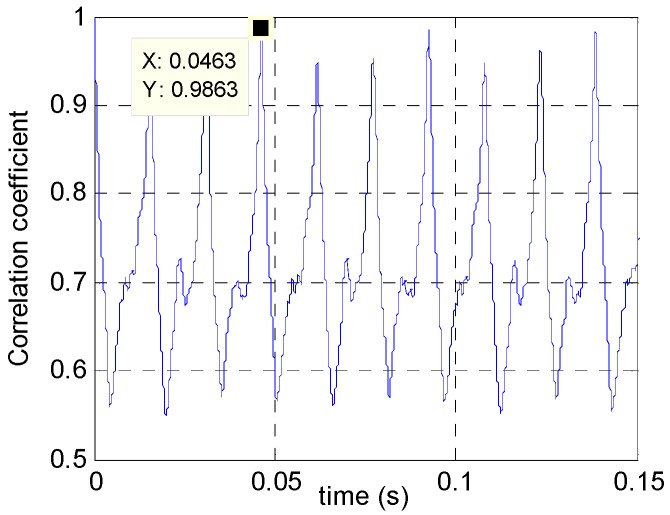
Estimated rotation period of blades.

Figure 20 shows the correlation coefficient after image registration on Figure 19 by Equation (16). With a template size of 5 × 5, it is found that blade recurring cycle is 0.0463 s and the rotation frequency is 22 per second. After processing the frequency value using the method reported in [2], the number of available blades is estimated at three, consistent with the actual number. For comprehensive validation, the same experiment and method were applied on different blades, all resulting in accurate estimation of the blade number *n*.

## 5. Discussion

This paper proposes a STFRF-based time-frequency algorithm. This new algorithm increases the signal length in the analysis window and enhances target SNR and frequency resolution. To overcome the heavy computation load associated with the conventional STFRF, this paper proposes an order prediction technique that makes use of signal continuity subsisting within a short time, thus improving the adaptability of the proposed technique. The analysis of a rotor helicopter indicates that, when the number of blades is two, the blade number can be accurately determined from STFRFT time-frequency image. When the number of blades exceeds two, it would be difficult to determine the number of blades directly from STFRFT time-frequency image, due to the presence of multiple time-frequency spectrum lines. This paper suggests using image registration technique to estimate the recurrence period of a same blade as well as the number of blades from STFRFT domain time-frequency spectrum. Experiment data validated the effectiveness of this technique in determining the period and number of blades of a multiple-blade target.

This paper proposes and verifies the characteristics and advantages of STFRFT. However, it should be noted that STFRFT transformation requires that the windowed signals must approximately be LFM signals. In practical radar applications, the signal sampling time might be short and the window length might be limited and this would reduce STFRFT’s advantages. For future work, a super-resolution spectrum estimation technique may be investigated to further improve STFRFT’s performance in time-frequency analysis.

## Figures and Tables

**Table 1:** Comparison of STFT and STFRFT time-frequency resolutions.

Time Point (s)	A(0)	B(0.2)	C(0.4)	D(0.6)	E(0.8)
Order of matching	0.99	1.0	1.02	1.03	1.05
STFT	1.0094	0.9520	1.1650	1.3858	1.9287
STFRFT	0.9520				
Time-frequency resolution ratio	1.1	1.1	1.4	1.7	2.3

**Table 2:** Comparison of order searching times consumed by different detection techniques.

SNR	Technique 1	Technique 2	Technique 3	T2/T1	T3/T1
−4 dB	18,0096	11,340	11,310	15.90	15.88
−6 dB	18,0096	12,430	12,400	14.52	14.48
−8 dB	18,0096	13,570	13,540	13.33	13.27

Technique 1 is the exhaustive search approach described in Ref. [26], Technique 2 and 3 are Equation (9) with an *L* of 2 and 3, respectively. T2/T1 represents the ratio between Technique 2 and Technique 1; T3/T1 represents the ratio between Technique 3 and Technique 1.

**Table 3:** Major parameters of radar system.

Parameter	Value
Carrier frequency	3 GHz (continuous wave)
Baseband sampling rate	78 kHz
Frame signal accumulation time	72.1 ms
Target	Rocket projectile

**Table 4:** Comparison of STFT and STFRFT.

Parameter	Time-Frequency Resolution Ratio
A	1.27
B	1.25
C	1.23

**Table 5:** Main parameters of acquisition system.

Parameter	Value
Frequency	674 MHz
Baseband sampling rate	5 kHz
Signal accumulation time	1 s
Target	Align 750e

**Table 6:** Main parameters of radar system.

Parameter	Value
Frequency	3 GHz (continuous wave)
Baseband sampling rate	20 kHz
Accumulation time	0.15 s
Target	Fan
